# Diagnostic value of presepsin in odontogenic infection: a retrospective study

**DOI:** 10.1186/s40902-022-00353-7

**Published:** 2022-06-06

**Authors:** Eun-Sung Kang, Jae-Hoon Lee

**Affiliations:** Department of Oral and Maxillofacial Surgery, College of Dentistry, Dankook University, 119 Dandae-ro, Cheonan, Chungcheongnam-do 31116 Republic of Korea

**Keywords:** Presepsin, Infection, SIRS, Sepsis

## Abstract

**Background:**

Most head and neck infections originate from odontogenic causes; therefore, it is important to determine the severity of odontogenic infections. Since severe infection can cause sepsis, a systemic examination should be performed when evaluating a patient with odontogenic infection. C-reactive protein (CRP), white blood cell count (WBC), procalcitonin (PCT), and presepsin (PSEP) can be used to evaluate the severity of inflammatory status and sepsis in patients in the early stages of visiting the emergency room. Moreover, sepsis can be diagnosed based on the systemic inflammatory response syndrome (SIRS) classification. In relation to PSEP, significant study results on sepsis have been reported in other organ infections. However, there has been no progress in odontogenic infection; therefore, this study aimed to determine the diagnostic value of sepsis derived from odontogenic infection.

**Methods:**

This study was conducted from March 2021 to October 2021 on 43 patients admitted to the Department of Oral and Maxillofacial Surgery, Dankook University Hospital, in the emergency room for odontogenic infection. All patients underwent vital sign assessment and diagnostic tests (CRP, WBC, PCT, PSEP) in the emergency room. Sepsis was classified according to the SIRS criteria, and CRP, WBC, PCT, and PSEP levels were measured. The Statistical Package for the Social Sciences was used for statistical analyses.

**Results:**

The results of this study showed a moderately positive correlation between CRP and PCT, CRP and PSEP, and CT and PSEP levels. In addition, PCT and PSEP levels showed a positive correlation with sepsis. The odds ratios of sepsis and PCT and sepsis and PSEP were statistically significant. The optimal cut-off values obtained through the receiver operating characteristic curve were 0.24 and 671.5 for PCT and PSEP, respectively. Finally, there were positive correlations between CRP level and length of stay, WBC and Flynn scores, PCT level and Flynn scores, PCT level and length of stay, and PSEP level and length of stay.

**Conclusion:**

WBC and CRP and PCT levels have been used in the past to determine the severity of infection and sepsis in patients with odontogenic infection, but PSEP was also found to have diagnostic value in this study. According to this study, a PSEP level of 671.5 pg/ml or higher for odontogenic infection can be considered an abnormal level.

## Background

Odontogenic infection is a type of bacterial infection that can appear in the maxillofacial region. When it is affected by the fascial spaces, rather than an infection limited to the teeth and alveolus level, it can cause cavernous sinus thrombosis and sepsis, including facial and cervical necrotizing fasciitis. Sepsis can lead to systemic tissue damage, organ failure, and death. Therefore, early diagnosis of sepsis is significantly important to predict a better prognosis [1–4]. Hence, for the diagnosis of sepsis, the SEPSIS-2 definition of systematic inflammatory response syndrome (SIRS) can be applied. The SIRS criteria include the following: (1) body temperature above 38 °C or below 36 °C, (2) heart rate > 90 beats per minute, (3) respiratory rate > 20 breaths per minute, and (4) white blood cell count (WBC) above 12,000 cu/mm or less than 4000 cu/mm. If two or more of these criteria are met and the infection is caused by bacteria, sepsis can be diagnosed [5, 6]. Diagnostic laboratory tests may be used as an adjunct to determine the severity of the patient’s inflammatory and infectious conditions. WBC count, C-reactive protein (CRP), procalcitonin (PCT), which are traditionally used, and presepsin (PSEP) can also be used [7, 8].

PSEP is a fragment of CD14, and CD14 is a cell surface antigen cluster marker protein expressed in bone marrow cells, observed on the surface of monocytes and neutrophils, and acts as a specific high-affinity receptor for lipopolysaccharide (LPS). After binding to LPS, it undergoes various signal transduction pathways and immune chain reactions [9]. There are two types of CD14, membrane-bound CD14 and soluble CD14 (sCD14). The plasma concentrations of sCD14 are elevated in certain diseases, such as sepsis, acquired immunodeficiency syndrome, acute respiratory distress syndrome, and systemic lupus erythematosus [8]. The immune response occurs when various pathogen-associated molecular patterns come into contact with the pathogen, where sCD14 acts as a ubiquitous correceptor, but sCD14 itself is not a specific factor for sepsis caused by bacterial infection [10]. Therefore, during the reaction of CD14, only the sCD14 subtype, a fragment of CD14 that binds to a bacterial pathogen specific for bacterial infection and is freed from the body’s circulation, was separately detected, and a specific marker for bacterial infection was discovered [8]. The epidemiological and biological activities of PSEP have not yet been clearly elucidated [10]. Enzyme immunoassay (EIA) was performed to measure the sCD14 subtype in plasma, and it was named PSEP.

In patients with sepsis, the plasma concentration increases faster than CRP or PCT level, and when EIA is performed, PSEP can be detected within 4 h of infection. Moreover, the examination time was significantly short (20 min) [8, 11]. According to studies evaluating the diagnostic value of the existing diagnostic tests for sepsis, PCT has a higher diagnostic value than the traditional diagnostic tests, such as CRP, WBC, and interleukin (IL)-6 [7]. Moreover, according to previous studies conducted on odontogenic infection, PCT plasma concentrations were higher than normal in patients with sepsis caused by odontogenic infection [12, 13]. However, PCT levels can be elevated by non-bacterial infections, such as burns, trauma, major surgery, intestinal ischemia, pancreatitis, cerebral hemorrhage, shock, and renal failure. Therefore, concurrent use of other diagnostic tests can increase the accuracy of the diagnosis of sepsis. In previous studies related to PSEP, the results of studies related to pneumonia, abdominal infection, and urethral infection showed that the higher the severity of sepsis, the higher the PSEP value [14–16].

As such, studies have shown that PSEP has high diagnostic value in diagnosing sepsis caused by various organ infections. However, as no studies related to odontogenic infection have been conducted, this study aimed to determine whether it can be applied to the field of odontogenic infection and whether it has sufficient diagnostic value for evaluating sepsis.

## Methods

### Study design and participants

From March 2021 to October 2021, among the patients who visited the emergency department of Dankook University Hospital, 43 patients were admitted to the Department of Oral and Maxillofacial Surgery for odontogenic infection. Among the patients with head and neck infections who visited the emergency room, only those with odontogenic infections were selected, and patients with other infections, such as salivary gland infections, sinusitis, osteomyelitis, drug-induced osteonecrosis, trauma, and secondary infections due to tumors, were excluded from this study.

If the patients with odontogenic infections met the following two or more SIRS criteria, they were classified into the sepsis group: (1) body temperature greater than or equal to 38 °C or less than 36 °C, (2) heart rate greater than 90 beats per minute, (3) respiratory rate greater than 20 breaths per minute, and (4) WBC greater than or equal to 12,000 cu/mm or less than 4000 cu/mm. The remaining patients were classified into the non-sepsis group.

### Test methods

As soon as the patients came to the emergency room, vital signs were measured, CRP level and WBC were measured to determine the inflammatory status, and PCT and PSEP levels, which are sepsis-related markers, were additionally measured. Dental panoramic radiography was performed to infer the source of the infection, and contrast-enhanced computed tomography was performed to identify the affected fascial spaces. The severity score (Flynn score) suggested by Flynn was used to evaluate the severity of the affected fascial spaces and infection severity [17].

After hospitalization, CRP level (mg/dL) and WBC (cu/mm) were measured daily until discharge, and PCT and PSEP levels were measured every other day until both values returned to normal. PCT (ng/mL) and PSEP (pg/mL) levels; the length of time taken for both diagnostic test values to be within the normal range, and the patient’s total hospitalization period were evaluated.

### Statistical analyses

Based on the collected patient group data, the correlation between diagnostic test values, difference in correlation between diagnostic test values depending on the presence of sepsis, and risk factors and cut-off values for diagnosing sepsis were analyzed.

Spearman correlation analysis was performed to investigate the correlation among diagnostic test values. The Mann–Whitney *U* test was performed to determine whether there was a difference in the diagnostic test values between the sepsis and non-sepsis groups. Using the binary logistic regression test, among the diagnostic test values, long-term hospitalization for more than 10 days and the risk factors of being diagnosed with sepsis were investigated, and the odds ratio was calculated. The receiver operating characteristic (ROC) curve was assessed to obtain the optimal cut-off value for diagnosing sepsis. Spearman correlation analysis was performed to examine the correlation among diagnostic test values, severity of the affected myofascial gap, and length of hospital stay. The sensitivity and specificity between PCT and PSEP were evaluated. The Statistical Package for the Social Sciences (SPSS) (SPSS version 27, IBM, New York, USA) was used for statistical analyses, and P values less than 0.05 were considered statistically significant.

## Results

### Participants

A total of 43 patients were recruited, of whom 22 were men and 21 were women. The mean age was 55.4 years. There were 24 and 19 patients in the non-sepsis and sepsis groups, respectively (Table 1).

**Table 1:** Characteristics of study subjects (group 1: sepsis)

Number	Sex	Age	CRP	WBC	PCT	PSEP	Flynn score	Hospitalization day
1	M	38	12.05	24,860	0.33	744	6	9
2	M	56	3.56	7020	0.12	738	2	13
3	F	86	29.48	17,750	10.8	2911	8	23
4	M	61	10.23	10,910	0.51	673	4	9
5	M	52	8.51	16,880	0.11	551	4	7
6	F	8	3.22	12,830	0.26	352	3	6
7	M	53	6.67	6920	0.96	692	4	11
8	F	73	24.9	19,770	24.7	730	14	19
9	M	45	5.15	13,060	1.12	387	3	14
10	F	7	5.44	14,620	0.91	330	7	4
11	F	58	16.99	12,240	0.88	707	10	14
12	M	74	17.26	8940	1.26	1998	1	13
13	M	80	21.02	11,720	0.51	719	9	10
14	F	72	31.87	17,780	0.26	944	2	12
15	F	46	6.11	20,500	0.73	616	6	12
16	M	44	11.17	18,630	0.14	479	13	13
17	F	63	2.68	21,280	0.07	974	7	6
18	M	44	21.45	9640	5.05	1276	14	22
19	M	34	19	13,900	0.93	582	8	13

Diagnostic test values, Flynn scores, and average, minimum, and maximum values for the hospitalization period were summarized. In the entire group, the mean CRP level, WBC, PCT level, and PSEP level were 11.4 mg/dL, 13514.2 cu/mm, 1.3 ng/mL, and 598.9 pg/mL, respectively. In the sepsis group, the mean CRP level, WBC, PCT level, and PSEP level were 13.5 mg/dL, 14697.4 cu/mm, 2.6 ng/mL, and 863.3 pg/mL, respectively. In the non-sepsis group, the mean CRP level, WBC, PCT level, and PSEP level were 8 mg/dL, 12577.5 cu/mm, 0.2 ng/mL, and 389.6 pg/mL, respectively. The maximum values were 32.61 mg/dL, 25490 cu/mm, 24.7 ng/mL, and 2911 pg/mL for CRP, WBC, PCT, and PSEP, respectively. Moreover, the minimum values were 1.56 mg/dL, 6920 cu/mm, 0.01 ng/mL, and 138 pg/mL for CRP, WBC, PCT, and PSEP, respectively. The mean Flynn scores were 5.0, 6.6, and 3.7 in the entire, sepsis, and non-sepsis groups, respectively. The average length of hospitalization was 10.2 days, with 12.1 and 8.7 days in the sepsis and non-sepsis groups, respectively. In the entire group, 15 and 28 patients had elevated PCT and PSEP levels above the normal range, respectively. In the sepsis group, 10 and 14 patients had elevated PCT and PSEP levels, respectively. In addition, in the sepsis group, 10 patients had elevated levels of both PCT and PSEP diagnostic tests (Tables 1, 2, and 3).

**Table 2:** Characteristics of study subjects (group 2: non-sepsis)

Number	Sex	Age	CRP	WBC	PCT	PSEP	Flynn score	Hospitalization day
1	F	48	10.62	13,230	0.05	236	2	12
2	F	31	5.74	10,000	0.04	327	1	5
3	M	60	15.14	7850	0.89	650	3	8
4	F	29	8.6	11,430	0.09	460	4	7
5	M	74	2.61	13,610	0.21	509	6	12
6	F	62	10.1	11,130	0.13	472	4	6
7	F	78	4.52	9980	0.07	670	2	8
8	M	56	4.33	25,490	0.04	250	9	7
9	M	57	9.41	12,240	0.29	379	6	9
10	F	35	2.94	20,860	1.44	306	4	9
11	M	49	1.56	9280	0.01	145	2	6
12	F	80	22.86	11,100	0.22	555	4	11
13	F	87	32.61	15,370	0.21	390	2	17
14	M	62	2.92	12,400	0.11	532	6	13
15	M	87	12.33	13,000	0.06	601	2	7
16	F	66	9.95	11,590	0.12	327	2	5
17	F	73	7.76	16,960	0.13	423	4	7
18	M	33	16.03	11,210	0.13	324	6	10
19	F	35	11.25	11,430	0.15	229	4	9
20	F	22	4.13	9690	0.05	254	2	5
21	M	33	7.52	7570	0.06	287	6	8
22	M	65	10.88	17,120	0.13	614	3	12
23	F	85	14.91	11,360	0.08	138	2	5
24	F	80	5.32	7960	0.05	272	2	11

**Table 3:** Mean values of diagnostic lab tests

Group	CRP	WBC	PCT	PSEP	Flynn score	Hospitalization
Total	11.4	13,514.2	1.3	598.9	5.0	10.2
Sepsis	13.5	14,697.4	2.6	863.3	6.6	12.1
Non-Sepsis	9.8	12,577.5	0.2	389.6	3.7	8.7

### Statistical analyses

#### 1) Correlation among diagnostic test values

The correlation among diagnostic values was investigated through the Spearman correlation analysis. CRP and PCT (0.443), CRP and PSEP (0.386), and PCT and PSEP (0.548) showed a positive correlation (*P* < 0.05) (Table 4).

**Table 4:** Correlation between diagnostic biomarkers

Group	Initial CRP	Initial WBC	Initial PCT	Initial PSEP
Initial CRP	1			
Initial WBC	0.65	1		
Initial PCT	0.443**	0.181	1	
Initial PSEP	0.386*	0.110	0.548**	1

p^*^ < 0.05, p^**^ *< 0.01*

#### 2) Differences in diagnostic test values according to sepsis

Based on the result of the Mann–Whitney *U* test, only PCT and PSEP showed statistically significant differences between the sepsis and non-sepsis groups (*P* < 0.05) (Table 5).

**Table 5:** Correlation between diagnostic biomarkers and sepsis

Group	Sepsis (*n* = 19)	Non-sepsis (*n* = 24)	*U*	*Z*	*P* value
Initial CRP	473	473	173	− 1.35	0.179
Initial WBC	479.5	466.5	166.5	− 1.5	0.133
Initial PCT	578.5	367.5	67.5	− 3.93	< 0.01
Initial PSEP	593	353	53	− 4.28	< 0.01

#### 3) Risk factors for sepsis and long-term hospitalization and odds ratio

PSEP and WBC underwent log2 conversions to adjust units similar to those of CRP and PCT. As the average length of hospitalization of the recruited patients was 10.7 days, the standard of long-term hospitalization was set at 10 days. Analysis of the results of the binary logistic regression test showed that PCT and PSEP levels were the only risk factors for the diagnosis of sepsis, and as these values increased, the risk of sepsis increased. The odds ratios of PCT and PSEP were 14.75 and 31.17, respectively. When the PCT level increased by 1, the risk of sepsis increased by 14.75 times, and when the Log2PSEP value increased by 1, the risk of sepsis increased by 31.17 times. The results were statistically significant. Regarding the risk factors for long-term hospitalization, none of the diagnostic test values showed statistical significance (*P* < 0.05) (Table 6).

**Table 6:** Odds ratio

	Sepsis		Hospitalization	
	*B* (SE)	OR	*B* (SE)	OR
Initial CRP	− 0.55 (0.07)	0.95	0.11 (0.6)	1.12
Log WBC	1.451 (1.16)	4.27	− 0.57(0.8)	0.57
Initial PCT	2.69 (1.22)*	14.75	0.83 (0.99)	2.3
Log PSEP	3.44 (1.37)*	31.17	0.83 (0.6)	2.3

p*<0.05

#### 4) Receiver operating characteristic curve and optimal cut-off value

**Fig. 1:**
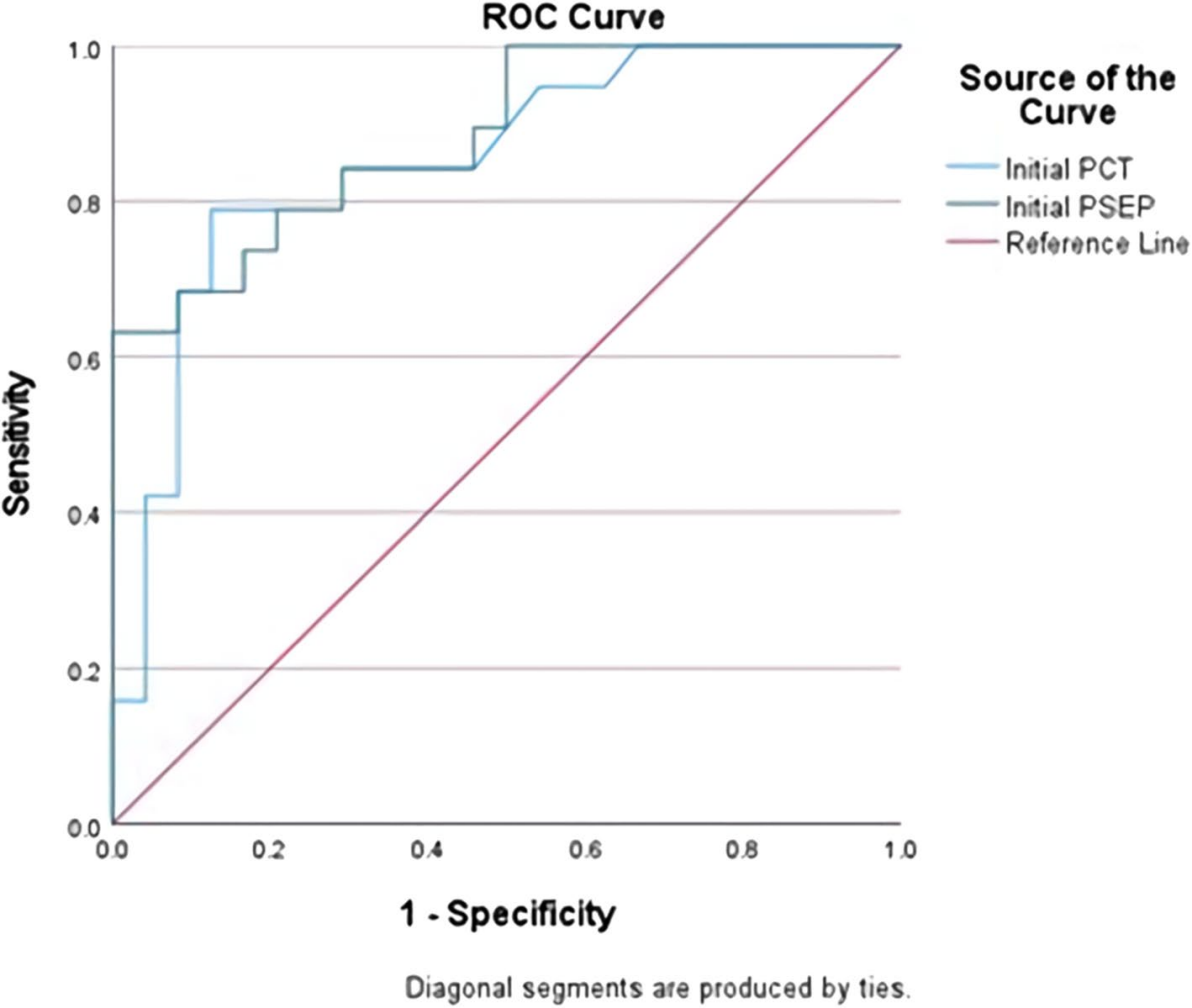
ROC Curve of PCT and PSEP

The ROC curves for PCT and PSEP were drawn. The AUCs for PCT and PSEP were 0.85 and 0.88, respectively, and the optimal cut-off value was calculated using the Youden index. The optimal cut-off value for the diagnosis of sepsis in PSEP was approximately 671.5 (Fig. 1) (Table 7).

**Table 7:** AUC

Findings	AUC	95% confidence interval	*P* value
Initial PCT	0.852	0.735–0.969	0.00
Initial PSEP	0.884	0.785–0.983	0.00

#### 5) Correlation among diagnostic test values, flynn scores, and length of hospitalization

The correlation among diagnostic test values, Flynn scores, and length of hospitalization was identified through the Spearman correlation analysis. The correlation scores between WBC and Flynn score and PCT level and Flynn score were 0.42 and 0.43, respectively, indicating a moderately positive correlation. The correlation scores between CRP level and length of hospitalization, PCT level and length of hospitalization, and PSEP level and length of hospitalization were 0.44, 0.55, and 0.5, respectively, showing a moderately rpositive correlation (*P* < 0.05) (Tables 8 and 9).

**Table 8:** Cut-off value

Findings	Cut-off value	Sensitivity	Specificity
PSEP	671.5	63%	100%

**Table 9:** Correlation between biomarkers and Flynn score/hospitalization day

	Flynn score	Hospitalization day
Initial CRP	0.16	0.44**
Initial WBC	0.42**	0.135
Initial PCT	0.43**	0.548**
Initial PSEP	0.284	0.502**

p* < 0.05, p** < 0.01

#### 6) Sensitivity and specificity

The sensitivities and specificities of PSEP and PCT measurements were 78.95% and 63.16% and 70.83% and 91.67%, respectively.

## Discussion

Odontogenic infections, the most common cause of bacterial infections in the maxillofacial region, rarely progress to sepsis. However, the possibility of progression to sepsis cannot be excluded in cases of moderate or severe infections affected by the fascial spaces [1, 2, 18, 19]. In this study, 19 patients with sepsis according to the SIRS criteria among the 43 recruited patients (approximately 44.2%) were classified as having sepsis.

According to a previous study assessing the diagnostic value of PSEP, Klouche et al. [14] stated that the plasma concentration of PSEP was significantly effective in the diagnosis of sepsis caused by pneumonia, moderate sepsis, and septic shock. According to a study by Vodnik et al. [15] on abdominal infections, higher levels of PSEP were measured in the sepsis group than in the SIRS and normal groups. According to a study by Sekine et al. [16] on urethral infection, PSEP was high in patients with septic shock, and their multiple logistic regression test results showed that it was a risk factor for sepsis. The results of the binary logistic regression test in this study showed that the risk of sepsis increased as the level of PSEP increased. In addition, as the odds ratio of Log2PSEP for sepsis was 31.17, significant results were also observed in the area of odontogenic infection.

According to a study by Kim et al. [13] related to the efficacy of PCT in odontogenic infection, the mean values were 7.24 and 0.40 in the sepsis and non-sepsis groups, respectively. In this study, the mean values were 2.6 and 0.2 in the sepsis and non-sepsis groups, respectively. Kim et al. [13] suggested that a PCT test should be performed concurrently with traditional diagnostic tests alone because the accuracy of sepsis due to maxillofacial infection is low. Considering the fact that the odds ratio of PCT in this study was 14.75, it is similar to that of Kim et al. In this study, the areas under the curve (AUCs) of the ROC curve were 0.927 and 0.85 in the sepsis and non-sepsis groups, respectively, which are relatively lower than those in previous studies. However, these values were statistically significant in this study and showed a value of 0.8 or higher, indicating that it is a good test method.

Comparing the accuracy and effectiveness among diagnostic test values, the PSEP level was greater than 110 ng/ml in patients with septic shock, and the optimal cut-off value was set to 110 ng/ml. At this time, the sensitivity and specificity were 0.727 and 0.617, respectively. In this study, sepsis was diagnosed based on the Sequential Organ Failure Assessment Score, and when multivariate analysis was performed, the odds ratios of PSEP and PCT were 0.0027 and 0.0024, respectively, which were statistically significant, whereas the odds ratio of CRP was not significant [20]. Zou et al. [21] compared PSEP, PCT, IL-6, and CRP levels, and when they compared the AUCs of their ROC curves, the following results were obtained: 0.845, 0.652, 0.672, and 0.815, respectively. When the optimal cut-off value of PSEP was set to 399 pg/ml, the sensitivity and specificity were 0.803 and 0.785, respectively, and when the optimal cut-off value was set to 600 pg/ml, the sensitivity and specificity were 0.878 and 0.814, respectively. According to a meta-analysis by Zhang et al. [22], the AUC and odds ratio of the ROC curve was 0.89 and 3.8, respectively. According to the meta-analysis of Wu et al. [23], the sensitivity and specificity of PSEP ranged from 0.67 to 1.0 and 0.33 to 0.98, respectively, according to the literature. The AUC and odds ratio were 0.88 and 16, respectively. The median cut-off value in the literature included in this study was 600 (439–664) pg/ml. According to a systematic review by Wu et al. the sensitivity and specificity of PSEP were 0.78 (0.76–0.80) and 0.83 (0.80–0.85), respectively. Moreover, the odds ratio and AUC of the ROC curve were 21.73 (12.81–36.86) and 0.89 (0.84–0.94), respectively. In this study, the AUC of PSEP was 0.88. This is similar to those of studies that have analyzed the diagnostic value of PSEP for sepsis in other organs. In addition, the optimal cut-off value in this study was 671.5, which was higher than the values set in the studies of Zou et al. [21] and Zhang et al. [22]. Based on the data collected in this study, the sensitivity and specificity of PSEP were 78.95% and 70.83%, respectively. The sensitivity was similar to those of other studies, but the specificity was low. The odds ratio of PSEP was 31.17, and PSEP was highly evaluated because its unit was reduced by converting the log.

Blood culture is important in diagnosing sepsis; however, it takes 48–72 h to confirm the sample result, and the probability of being positive is also low. The plasma concentration of PCT increased 4 h after infection and increased slowly over 8–24 h, reaching a maximum concentration 1 day after infection [21]. However, PSEPs are detectable at an earlier time. The concentration increased within 2 h and reached a peak within 3 h. In addition, the time required for the results to appear was significantly short (approximately 20 min) [11]. Therefore, it is reasonable to test for PSEP in the early diagnosis of sepsis in the emergency room. According to Ulla et al. [24], the plasma concentrations of PSEP and PCT were increased in patients with sepsis who visited the emergency room. The PCT and PSEP levels were 0.875 and 0.701, respectively, and PCT had a high AUC, but the mean PSEP was higher in patients who died within 60 days of hospitalization due to sepsis.

The Flynn score was used to evaluate the severity of odontogenic infections in hospitalized patients [17]. In this study, the affected fascial and diagnostic test values with statistical significance were WBC and PCT. However, because the Flynn score only evaluates the affected fascial spaces, it relatively insufficiently evaluates systemic infection.

This study has a limitation in that the number of patients was insufficiently large.

## Conclusions

PCT, CRP, and WBC, which are traditional diagnostic tests, have been used in patients with odontogenic infections. However, according to the results of this study, PSEP can also be useful in determining the severity of odontogenic infection and sepsis, and when combined with existing test methods, it is expected to be better in evaluating patient prognosis.

## Data Availability

The datasets used and/or analyzed during the current study are available from the corresponding author on reasonable request.

## Abbreviations

SIRS: Systemic inflammatory response syndrome
CRP: C-reactive protein
WBC: White blood cell count
PCT: Procalcitonin
PSEP: Presepsin
ROC: Receiver operating characteristic
AUC: Area under the curve
